# Assessment of biphasic calcium phosphate 70/30 alginate scaffold on the tibia in pigs

**DOI:** 10.14202/vetworld.2020.2635-2642

**Published:** 2020-12-11

**Authors:** Gunanti Soeyono, Kiagus Dahlan, Melpa Susanti Purba, Sus Dherthi Widhyari, Rr. Soesatyoratih, Thang Shi Teng, Lieonny Budiarti, Ho Kin Wai, Agatha Kosat

**Affiliations:** 1 Department of Veterinary Clinic Reproduction and Pathology, Division of Veterinary Surgery and Radiology, Faculty of Veterinary Medicine, Bogor Agricultural University, Bogor, Indonesia; 2 Veterinary Paramedic Study Program, Vocational School, Bogor Agriculture University, Bogor, Indonesia; 3 Department of Physics, Faculty of Mathematics and Natural Sciences, Bogor Agricultural University, Bogor, Indonesia; 4 Department of Veterinary Clinic Reproduction and Pathology, Division of Veterinary Medicine, Faculty of Veterinary Medicine, Bogor Agricultural University, Bogor, Indonesia

**Keywords:** biphasic calcium phosphate, bone implant, erythrogram, pig, pulmonary radiography, scaffold

## Abstract

**Background and Aim:**

Calcium phosphate bioceramics have been used for at least a decade, and many investigations have focused on the use of hydroxyapatite (HA) derivative in the regeneration of bone defects. Biphasic calcium phosphate (BCP) is a biomaterial composed of HA and beta-tricalcium phosphate (BCP), with a structure similar to bone. The aim of the study was to determine the influence of the BCP/alginate scaffold on tissue growth, blood, the lungs, and the electrical activity of the heart during bone healing in the tibia of pig.

**Materials and Methods:**

Three pigs were implanted with BCP/alginate scaffolds in the tibias. Pigs were acclimatized and treated with antibiotics and anthelminthic drugs 14 days before implantation. Each pig was implanted with a BCP/alginate scaffold in the right tibia and a defect without the implant was made in the left tibia as the control. Radiographic images of the tibia were captured 0, 7, 30, and 60 days after the operation. Erythrograms, radiography of the lungs, and electrocardiogram (ECG) recordings were done 0, 30, and 60 days after the operation.

**Results:**

Radiographic evaluations showed that the implant and peri-implant density of BCP decreased throughout the process of bone healing. The erythrogram profile indicated that a substantial amount of time (60 days) was required to adapt and return to pre-operative conditions. No significant differences in ECG recordings or pulmonary radiography were detected.

**Conclusion:**

The BCP/alginate scaffold did not induce a faster recovery rate from the bone defect compared to the control with no implant. However, the BCP/alginate scaffold was biodegradable, bioresorbable, and non-toxic.

## Introduction

Bone implants and heterologous materials have been under development for many years to enhance the quality of life in humans after trauma, bone cancer, fractures, and osteoporosis. The use of biomaterials is increasing the focus of investigations. An ideal biomaterial must be biocompatible and have little effect on other parts of the body. In addition, biomaterials for bone implants should possess the following properties: Osteoinductivity to stimulate mesenchymal cell differentiation into bone-forming osteoblasts; osteoconductivity to serve as a matrix for vascular and cellular migration, and osteointegrative properties with osteoprogenitor cells to produce a new bone matrix [1]. To ensure safety, investigations into a new biomaterial must be repeated many times *in vitro* and *in vivo*. For this reason, animal models are important in developing new biomaterials before clinical applications in humans. Animals, such as dogs, sheep, goats, and pigs, are ideal animal models in the research of biomaterials. Animal models, such as pigs, are used to study medication treatment and efficacy. Pigs are used in many investigations because of similarities in their biomedical and hemodynamic characteristics to humans. The pig and human bone similarities include bone mineral density, anatomy, morphology, remodeling rate, and bone recovery [2-5]. The regeneration rate of bones is similar in pigs and humans; human bone regeneration is approximately 1.0-1.5 μm/day and pigs regenerate approximately 1.2-1.5 μm of bone per day [6,7]. Because of these similarities, pigs are being used increasingly to model different types of orthopedic surgical conditions [8].

Biomaterials are divided into several categories, including autograft, allograft, xenograft, and synthetic materials. In this study, bioceramic biphasic calcium phosphate (BCP) with a hydroxyapatite (HA) to beta-tricalcium phosphate ratio of 70:30 was used. This scaffold was chosen because of its good biocompatibility, biodegradability, and non-toxic characteristics [9,10]. The calcium phosphate in this experiment was extracted from eggshells and combined with alginate, a natural polymer extracted from brown seaweed.

The BCP scaffold in this research underwent surface porosification (200-400 μm), followed by shaping with freeze-drying. This study was conducted to determine the influence of a BCP/alginate scaffold implant in the tibia on tissue growth, blood, the lungs, and the electrical activity of the heart throughout the bone healing process.

## Materials and Methods

### Ethical approval

This research had received ethical approval from the Animal Ethics Commission of the Faculty of Veterinary Medicine, Bogor Agricultural University with the SKHE No. 052/KEH/SKE/I/2017.

### Study period and location

This research was conducted from January to April 2017. Blood collection and surgery at the Laboratory of the Surgery and Radiology Division, Department of Clinical, Reproductive and Pathology, Faculty of Veterinary Medicine, IPB University. Meanwhile, the maintenance of experimental animals was carried out in the cage of the Laboratory Animal Research Faculty of Veterinary Medicine, IPB University.

### Animals

Three male piglets (*Sus scrofa*), 2-3 months old, with body weights (BWs) of 20-25 kg, were used. The piglets underwent acclimatization in an animal cage at the Laboratory Animal Management Unit of the Faculty of Veterinary Medicine, Bogor Agricultural University. During the acclimatization process, the animals were fed twice a day and given drinking water *ad libitum*. Long-acting oxytetracycline (1 mL/10 kg BW) and anthelmintic oxfendazole (4.5 mg/kg BW) were administered orally. Animal cages were cleaned twice a day (morning and evening). The acclimatization period allowed the animals to adapt to the new environment before the beginning of the study [3].

### Procedures

**Figure-1:**
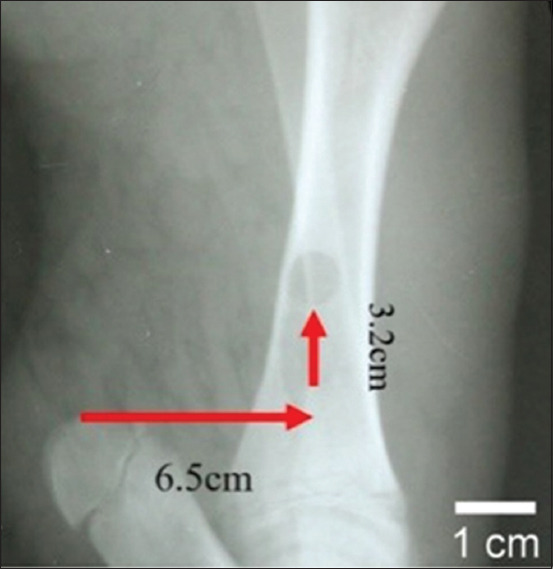
Incision at medial tibia with synchronized orientation of 6.5 cm horizontal from the calcaneal tuber and 3.2 cm vertical towards proximal.

Animals were sedated with a combination of 10% Ketamine HCl at 20 mg/kg BW and 2% Xylazine HCl at 2 mg/kg BW. Both drugs were given intramuscularly (IM) in the cervical trapezius muscle. All surgical procedures were performed under aseptic conditions. An incision was made medial to the tibia, 6.5 cm horizontal from the calcaneal tuber and 3.2 cm vertical in the proximal direction, as shown in Figure-1. Defects were drilled into both tibias to accommodate the bone-implant with a diameter and depth of 0.8 cm and 0.4 cm, respectively. The left tibias acted as the controls (without implants). BCP/alginate scaffoldings were implanted into the right tibias. Approximately 1 mL of a topical antibiotic (procaine benzylpenicillin, 50,000 IU/mL) was applied to the bone and muscle before surgical site closure. The same surgeon performed the operations in all animals to prevent defect variations. Povidone-iodine (3%) and Peru balsam were applied to the surgical site every day for a week to prevent post-operative secondary infection. The same antibiotic used in the acclimatization period was given IM and post-operative pain control was treated with flunixin meglumine IM (1.1-2 mg/kg once a day for 3 days). Post-operative care was performed as mentioned above. Post-operative physical examinations were conducted daily for 7 days to observe abnormalities, such as lameness, poor wound recovery, or changes in temperature, heart rate, and respiration rate. A daily evaluation was conducted manually and visually. The animals were euthanized on post-operative day 60.

### Radiographic assessments

**Figure-2:**
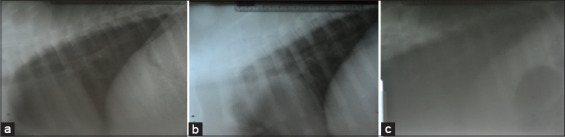
Laterolateral radiographic images (a) day 0, (b) day 30, (c) day 60.

Mediolateral and craniocaudal images of both tibias were taken to assess the healing process and interaction of the BCP/alginate scaffold with the surrounding tissues. Radiographic images of the tibia were taken preoperatively (15 min before surgery) and on post-operative days 0, 7, 30, and 60, with constant 58 kVp, 1.0 mAs, and a 40-inch focal film distance (FFD). All pigs were in the left lateral recumbent position for laterolateral radiographic images of the lungs with constant 70 kVp, 1.2 mAs, and a 40-inch FFD. The pulmonary evaluation was performed based on changes between pre- and post-operative radiographic images (pulmonary are clean) (Figure-2). X-ray films were processed manually in the darkroom, dried with a blow dryer, and interpreted using an illuminator. Photographs of the radiography images were taken with a digital single-lens reflex camera, and the bone density was evaluated with Image-J^®^ software (https://imagej.nih.gov/ij/download.html).

### Blood samples

Blood was collected preoperatively from the superior vena cava into a tube containing ethylenediaminetetraacetic acid. Blood samples were taken again on post-operative days 30 and 60. The samples were sent to one of the commercial blood laboratories in Bogor for analyses.

### Electrocardiogram (ECG)

ECG recordings of all animals were taken in a sedated condition. ECGs were recorded with the animal in the lateral recumbent position using an electrocardiograph (Fukuda ME Cardiosunny D300^®,^ USA) calibrated at 1 mV = 10 mm, with a rate of 50 mm/s. ECGs were recorded preoperatively (5 min before surgery) and on post-operative days 0, 30, and 60. Three bipolar (leads I, II, and III) and three unipolar (leads aVR, aVL, and aVF) ECG electrode leads were used. Four electrodes, clipped on the right and left front limbs, and right and left hind limbs, were used (red, yellow, green, and black). The amplitude, duration of P wave, PR interval, QRS interval, and amplitude and duration of the R and T waves were determined.

### Statistical analysis

The statistical analysis was performed using SPSS Version 16 (IBM, USA) software. Quantitative data were analyzed using analysis of variance and Duncan’s multiple range tests. The results are presented as the mean ± standard deviation or qualitatively in a narrative description. The radiographic images were analyzed using Image-J 3.82 version (https://imagej.nih.gov/ij/download.html).

## Results

**Figure-3:**
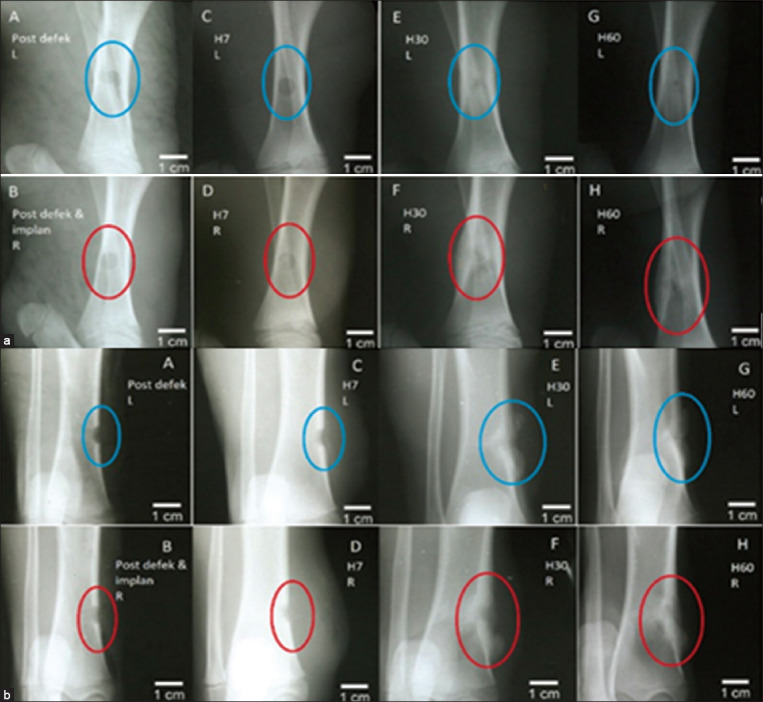
Radiographic images of the tibial bone (days-0, 7, 30, and 60) upper row-defect control, lower row-defect with implant. (a) Mediolateral image of tibial bone (b) craniocaudal image of tibial bone.

The defect made intentionally was measured by taking radiographic images that were analyzed using Image-J^®^. The defect sizes in the control and implants were 87.5 and 37.5 mm, respectively. The size of the defects changed little from days 0 to 7, but changed more drastically from days 7 to 30, as shown in Table-1 and Figure-3. Both mediolateral and craniocaudal radiographic images show a radiopaque sphere on the implanted tibia (shown by the circles in Figure-3), which represents the presence of the BCP/alginate scaffold. Both radiographic images showed no abnormal changes.

**Table-1:** Difference of defect size based on the time of interpretation and the decrease in defect size (%).

Defect	Days		Decrease in defect size (%)		
	
	0	7	30	60	
Defect with implant	8.0	7.3	5.5	5.0	37.5
Δ	-	0.7	1.8	0.5	
Defect control	8.0	7.0	3.0	1.0	87.5
	-	1.0	4.0	2.0	

Δ=Difference in size between the current defect and previous defect

Changes in the density of the radiolucent zone, margination, and shape of the bone are shown in Table-2. On day 30, the density of the radiolucent zone decreased and the density decreased even more by day 60. Changes in margination were similar between the defect control and implant, as shown in Table-3. Changes in margination started on day 30 and remained the same until day 60. Margination on the implant defect showed more changes than that on the control defect throughout the experiment.

**Table-2:** Degree of changes in radiolucent zone based on time of interpretation (days) with mediolateral and craniocaudal radiographic images.

Time of interpretation	Degree of changes in radiolucent zone			

	Mediolateral		Craniocaudal	
	
	DI	DC	DI	DC
D-0	+++	+++	+++	+++
D-7	+++	+++	+++	+++
D30	++	++	+	+
D-60	+	+	+	+

DI=Defect with implant, DC=Defect control

**Table-3:** Changes in bone margination based on time of interpretation (days) with mediolateral and craniocaudal radiographic images.

Time of interpretation	Changes in margination			

	Mediolateral		Craniocaudal	
	
	DI	DC	DI	DC
D-0	–	–	–	–
D-7	–	–	–	–
D-30	++	–	++	+
D-60	++	–	++	+

DI=Defect with implant, DC=Defect control

The density of both the defect control and biomaterial implant decreased on day 7, increased slightly on day 30, and decreased drastically on day 60, as shown in Table-4. The peri-implant density of the defect control and implant increased on day 7 and then decreased until day 60, as shown in Table-5. Both implant and peri-implant densities decreased overall until day 60.We made fracture from the beginning, which occurred in two pigs a few days after the operation, the radiographic data were taken from the remaining pigs.

**Table-4:** Radiographic density of implant (arbitrary unit) based on time of interpretation (days).

Time of interpretation	Density	

	DI	DC
D-0	92.00	84.08
D-7	82.31	55.77
D-30	84.40	67.69
D-60	47.41	48.31

DI=Defect with implant, DC=Defect control

**Table-5:** Radiographic density of peri-implant (arbitrary unit) based on time of interpretation (days).

Time of interpretation	Density	

	DI	DC
D-0	192.22	158.44
D-7	239.67	213.00
D-30	188.22	201.22
D-60	182.89	134.33

DI=Defect with implant, DC=Defect control

The normal erythrocyte range is 5-8 × 10^6^ μL, the normal hemoglobin concentration range is 10-16 g/dl, and the normal hematocrit range is 36-43% [12]. The surgery caused no distinctive changes in these parameters. Hemoglobin and hematocrit values increased on day 30 and decreased on day 60. The average erythrogram was lower than the normal range (Table-6).

**Table-6:** The average result of erythrogram.

Parameter	0^th^	30^th^	60^th^
RBC	3.26±0.23^a^	3.40±0.79^a^	2.93±0.40^a^
Hemoglobin	9.97±0.35^a^	10.03±0.66^a^	8.80±1.30^a^
Hematocrit	30.00±1.00^a^	32.33±2.52^a^	26.33±4.04^a^

Same superscripts

(a) in the same column indicate no significant differences of the PR intervals from different time of interpretation (p<0.05)

The ECG from the bipolar leads (II) was interpreted before and after surgery (days 0, 30, and 60) (Tables 7-11). As the P waves are usually most prominent in lead II, this lead was used to measure the amplitude and duration of the P waves [13]. The degree of atrial depolarization of the heart was determined by the P wave amplitude. The P wave duration was also measured to determine the time of atrial depolarization. The normal amplitude and duration of the P waves in piglets are 0.1-0.3 mV and 0.020-0.040 s, respectively [14]. The average amplitude and duration of the P waves after surgery were within the normal range.

**Table-7:** The average amplitude and duration of P wave of the pigs.

Time of interpretation	P wave	

	Amplitude (mV)	Duration (s)
Pre-implant (days-0)	0.07±0.12^a^	0.01±0.01^a^
Post-implant (days-0)	0.14±0.12^a^	0.02±0.02^a^
Days-30	0.06±0.02^a^	0.03±0.00^a^
Days-60	0.21±0.05^a^	0.04±0.00^a^
Normal range	0.1-0.3	0.020-0.040

Same superscripts

(a) in the same column indicate no significant differences of the PR intervals from different time of interpretation (p<0.05)

**Table-8:** The average of PR interval of the pigs.

Time of interpretation	PR interval (s)
Pre-implant (days-0)	0.03±0.06^a^
Post-implant (days-0)	0.08±0.07^a^
Days-30	0.12±0.01^a^
Days-60	0.12±0.03^a^
Normal range	0.06-0.13

Same superscripts

(a) in the same column indicate no significant differences of the PR intervals from different time of interpretation (p<0.05)

**Table-9:** The average QRS complex intervals of the pigs.

Time of interpretation	QRS complex interval (s)
Pre-implant (days-0)	0.04±0.01^a^
Post-implant (days-0)	0.05±0.01^a^
Days-30	0.03±0.00^a^
Days-60	0.04±0.00^a^
Normal range	0.030-0.050

Same superscripts

(a) in the same column indicate no significant differences of the PR intervals from different time of interpretation (p<0.05)

**Table-10:** The average amplitude and duration of R wave of the pigs.

Time of interpretation	R wave	

	Amplitude (mV)	Duration (s)
Pre-implant (days-0)	0.54±0.26^a^	0.01±0.01^a^
Post-implant (days-0)	0.61±0.19^a^	0.02±0.01^a^
Days-30	0.55±0.16^a^	0.02±0.01^a^
Days-60	0.60±0.09^a^	0.01±0.00^a^
Normal range	0.0-1.0	0.01-0.02

Same superscripts

(a) in the same column indicate no significant differences of the PR intervals from different time of interpretation (p<0.05)

**Table-11:** The average amplitude of T wave of the pigs.

Time of interpretation	T wave amplitude (s)
Pre-implant (days-0)	–0.00±0.00^a^
Post-implant (days-0)	0.15±0.25^a^
Days-30	0.10±0.16^a^
Days-60	–0.06±0.15^a^
Normal range	(–)/(+)

Same superscripts

(a) in the same column indicate no significant differences of the PR intervals from different time of interpretation (p<0.05).

The PR interval is the period that extends from the beginning of atrial depolarization until the beginning of ventricular depolarization. The normal PR interval in 2-4-month-old pigs is 0.06-0.1 s [15]. The average duration of the PR intervals (30 and 60 days) was shorter than the normal range. The shortened PR interval may be due to an impulse disturbance in the heart. Shortened PR intervals may also be caused by premature ventricular contractions [16].

The QRS complex interval represents the time of ventricular depolarization and indicates the ventricular function of the heart. The normal range for the QRS complex interval in piglets is 0.030-0.050 s [15]. The QRS complex intervals changed over time (p<0.05) after surgery. However, the average QRS complex interval was within the normal range.

The amplitude of the R wave represents the degree of ventricular depolarization, whereas the duration of the R wave represents the time of ventricular depolarization. The normal range of R wave amplitude in piglets is 0.0-1.0 mV [15]. The amplitude and duration of the R wave were within the normal range taken after surgery and in both groups.

**Figure-4:**
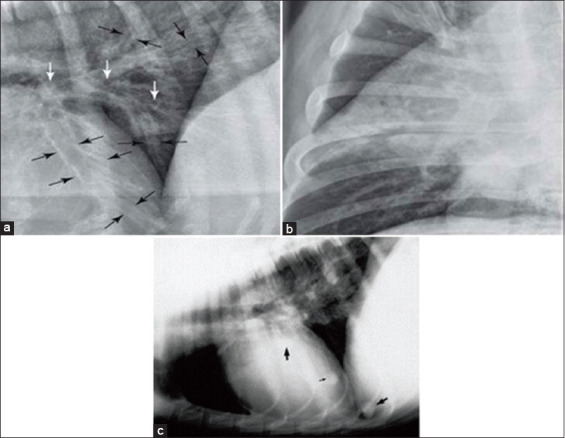
(a) Bronchial pattern [24], (b) lobar sign [24], and (c) cotton-like density [25].

The T wave on the ECG represents the ventricular repolarization phase of the heart after contraction. T waves are normally biphasic and positive on leads I, II, and III. Normal T waves in piglets can be positive or negative [15]. There was no change in T wave. Radiographic images were used to search for the presence of pulmonary abnormalities, such as pulmonary vein dilation, bronchial pattern, cotton-like density, and lobar sign. No signs of pulmonary abnormalities were observed after surgery in both groups, as shown in Figure-4.

## Discussion

Many parameters should be investigated repeatedly to ensure the safety of biomaterials, including their cytotoxicity, genotoxicity, and cell proliferation. Animal models are used widely in these investigations because they provide reliable preliminary information before continuing the research in humans. However, the differences between species provide both advantages and disadvantages. In this study, a piglet model was used to assess the efficacy and safety of BCP/alginate implants. In our results, it shows that there was no significant change in pulmonary radiography image and EKG evaluation. We used *Sus-scrofa* pigs as an animal model since it has similar anatomy and physiology as human.

The size of the tibia defect decreased on day 7, which coincides with the first phase of bone healing when inflammation occurs due to damaged bone tissues [17]. During the inflammation phase, immune cells, such as monocytes, lymphocytes, polymorphonuclear cells, and fibroblasts, play an important role in the bone healing process [18]. The second stage of bone recovery includes the growth of soft callus. In this experiment, the diameter of the defect decreased drastically on day 30 after the operation, coinciding with the second stage. In this period, the control defects recovered better than the defects treated with the BCP/alginate scaffolds. Implant defects had a higher density than control defects because of the presence of BCP alginate, which absorbs more X-rays than bone.

The density of both the implant and peri-implant decreased throughout the experiment. The decreased density may be due to the interactions between the implant and chronic inflammation, resulting in implant degradation. During this phase, the body responds to foreign material by protein deposition, coagulation, inflammation, and tissue growth [18]. Based on the change of density in this experiment, higher concentrations of calcium from the BCP/alginate scaffold may have created an apatite layer around the defect, resulting in decreased density [19]. For hip joint and femur fractures, the proper timing of the first radiograph is 6 weeks after the operation [20]. Several factors affect the density score, including the manual chemical process of the X-ray film and the stabilization of electric current during the X-ray exposure [21]. However, the mechanisms by which BCPs contributed to osteoconductivity and osteoinductivity at the molecular level are not understood fully [22].

There are several limitations to this study. Radiographic images could not be taken of the tibias that were fractured because of the presence of wires and pins that were attached to the bones. In addition, the implant size was too large for the corpus of the tibia in pigs. The age of the animal model might have contributed to the fractures; the injured limbs could not support the quickly increasing piglet weight. Severe hemorrhage during the surgery might reduce erythrocyte numbers and it can cause complications such as acute or chronic anemia [23]. Large red blood cell losses are an early sign of acute anemia. However, the fracture that occurred during this experiment could also have influenced the erythrocyte number. In this experiment, a longer time was required for the recovery of erythrocyte counts.

ECG results showed that the BCP/alginate tibial implant did not affect the electrical activities of pig hearts. No pulmonary abnormalities, such as pulmonary vein dilation, peribronchial pattern, cotton-like density, and lobar signs, were observed before or after implanting the scaffold. Thus, the BCP/alginate scaffold implant did not cause pulmonary abnormalities in this experiment.

## Conclusion

Bone recovery is detected as radiodensity. As shown on the X-rays, radiodensity was observed on the sides of the mediolateral and craniocaudal images, indicating that the implant material triggered more callus development than the control. No pulmonary abnormalities were found in response to implanting the BCP/alginate scaffolding material. Furthermore, cardiac parameters measured by the ECG were all within the normal range after implanting the BCP/alginate scaffold. The implanted BCP/alginate scaffold did not affect red blood cell, hemoglobin, and hematocrit values. In summary, while the BCP/alginate scaffold was not capable of inducing a faster recovery rate from the bone defect compared to the control with no implant, the BCP/alginate scaffold was non-toxic.

## Authors’ Contributions

GS and KD: Coordinator, background theory, design of experiment, methodology, data collection, data analysis and interpretation, writing and reviewing the text. SDW and RS: Background theory design of experiment, methodology, data collection, data analysis and interpretation, writing and reviewing the text. MSP, TST, LB, HKW, and AK: Data collection, data analysis and interpretation, writing and reviewing the text. All authors read and approved the final manuscript.
